# Beyond Iron Solubility: Particle Size as a Determinant of Cell Survival and Iron-Induced COX-2 Expression in Human Intestinal Cells

**DOI:** 10.3390/biom16030388

**Published:** 2026-03-05

**Authors:** Agata Tarczykowska, Amir Saeid Mohammadi, Nathalie Scheers

**Affiliations:** 1 Department of Life Sciences, Chalmers University of Technology, 41296 Gothenburg, Sweden; 2 Department of Architecture and Civil Engineering, Chalmers University of Technology, 41296 Gothenburg, Sweden

**Keywords:** ferric pyrophosphate, ferric EDTA, ferrous fumarate, COX-2, ferritin, solubility, Caco-2, Hutu-80

## Abstract

**Background:** Oral iron supplementation or food fortification is essential for managing or preventing iron deficiency but often causes gastrointestinal side effects. While solubility has traditionally been considered a requirement for iron uptake via the DMT1 transporter, recent evidence shows that insoluble iron can also be absorbed through endocytosis, raising questions about particle size and epithelial responses. **Methods:** Human intestinal cell lines (Hutu-80 and Caco-2) were exposed to physiologically relevant but elevated iron levels (0.5 mM Fe, 48 h) as ferric pyrophosphate, ferrous fumarate (both prone to precipitation), and soluble ferric EDTA. Cell survival and COX-2 protein were quantified by ELISA, solubility by ICP-OES, and particle size in cell culture medium by dynamic light scattering analyses. **Results:** Ferric pyrophosphate (0.62–3.8 μm) markedly increased COX-2 expression in Hutu-80 cells (254% ± 37%, *n* = 3, *p* = 4.11 × 10^−5^) and in Caco-2 cells (78% ± 8%, *n* = 3, *p* = 0.01) compared to the control. Ferrous fumarate (237–866 nm) also induced COX-2, but only in Hutu-80 cells (62% ± 11%, *n* = 3, *p* = 0.04), whereas ferric EDTA showed no effect in either cell line. COX-2 induction was associated with larger particles in the medium (≥237 nm), whereas smaller particles (<146 nm) were not. **Conclusions:** Particle size appears to be a critical determinant of cell survival and iron-induced epithelial COX-2 expression. Iron compounds that present as both soluble and particulate forms may optimize bioavailability, but controlling aggregate size (<146 nm) could reduce inflammatory signaling. These findings may have important implications for cell culture systems and warrant *in vivo* validation in iron supplemental studies.

## 1. Introduction

The chemical form of iron, whether a salt or a chelate, has significant implications for both its nutritional efficacy and safety profile [1]. Some iron salts, such as ferrous sulfate, dissociate rapidly and can lead to localized iron accumulation, resulting in higher reactive oxygen species (ROS) production as was observed in a study that compared HT29 cells to whey protein-based iron microspheres [2]. This pro-oxidative effect was not observed to the same extent with iron chelates such as iron polymaltose or ferric EDTA [3]. However, the iron chelates ferric EDTA and ferric citrate have been shown to promote colon cancer in mice [4,5,6], and cell studies confirmed their activation of oncogenic signaling via increased amphiregulin, EGFr, and ERK phosphorylation in Caco-2 and Hutu-80 cells, an effect not seen with ferrous sulfate [7]. Epidemiological data suggest that dietary heme-iron (a tetradentate iron chelate) intake of >1 mg/day raises colorectal cancer risk, while higher levels of iron storage biomarkers are linked to a decreased cancer risk [8].

Metal–ligand interactions, which play a central role in determining the bioavailability and physiological effects of iron supplements, are of crucial importance, particularly when comparing iron salts and chelated forms [1,7,9,10,11]. Luminal iron can, for example, exist as hydrated complex ions ${\left[Fe(H_{2}{O)}_{5}\right]}^{3+}$ and ${\left[Fe(H_{2}{O)}_{6}\right]}^{2+}$ [6], or as a part of complexes with organic or inorganic ligands, and the stability, solubility, and number of coordination sites of these complexes are critical factors that influence absorption in the gastrointestinal tract [12,13]. Iron administered as simple salts (e.g., ferrous fumarate and ferrous sulfate) or chelates (e.g., ferric pyrophosphate and ferric EDTA) exhibits different absorption profiles in the human gastrointestinal tract [9,14]. Ferrous iron (Fe^2+^) remains relatively soluble at both acidic and neutral pH in anoxic environments. However, in the presence of oxygen, as is the case in the duodenum and proximal jejunum, aqueous Fe^2+^ is rapidly oxidized to ferric iron (Fe^3+^) [15]. At neutral pH, Fe^3+^ is practically insoluble (solubility ~10^−18^ M). As the pH rises above 3.5 in the duodenum and proximal jejunum, the primary sites of non-heme iron absorption [16,17], ferric iron tends to precipitate as ferric oxide-hydroxide, unless stabilized by appropriate ligands [9,15]. Supporting this, Garcia-Casal et al. demonstrated that 74% of the iron salt ferrous fumarate precipitated within 10 min when the pH was increased from 2 to 6; at the same time, ferric EDTA stayed soluble in 95% [18].

Iron absorption in the intestine occurs through distinct pathways that depend, among other factors, on solubility. Soluble iron, primarily in the ferrous form (Fe^2+^), is transported across the apical membrane of enterocytes via the divalent metal transporter 1 (DMT1), following a reduction from ferric to ferrous iron by enzymes such as duodenal cytochrome b (Dcytb) [19,20]. In contrast, more recent studies indicate that insoluble iron species, including particulate or aggregated forms, can enter cells through endocytic processes, suggestive of clathrin-mediated endocytosis [21,22,23]. These two uptake mechanisms may operate simultaneously, potentially enhancing overall iron bioavailability, but they also raise important questions regarding epithelial responses to particulate iron and its safety implications.

### The Present Study

COX-2 is an inducible enzyme activated by inflammatory stimuli and has been independently associated with cancer-related inflammation and tumor progression [24,25,26,27]. In this study, we investigated the previously observed COX-2 induction by ferric pyrophosphate in the human duodenal Hutu-80 cell line [10], extending the analysis to include both Hutu-80 and Caco-2 intestinal cell lines and two additional iron forms: ferric EDTA and ferrous fumarate. Our earlier work demonstrated that ferric pyrophosphate and ferric EDTA, both iron chelates, elevate cellular amphiregulin levels; however, only ferric pyrophosphate induced COX-2 expression. The aim of the present study is, therefore, to explore the basis of this difference and to compare these chelates with ferrous fumarate, an iron salt that has not previously shown adverse effects in our experiments.

Comparisons were conducted at a high but physiologically relevant concentration (0.5 mM Fe), corresponding to an oral dose of approximately 170 mg iron in humans [28]. This dose was selected to enable a comparison with previous data. While lower doses may not induce COX-2 expression, the purpose here was to use a concentration sufficient to cause the COX-2 response, thereby allowing mechanistic investigation.

## 2. Materials and Methods

### 2.1. Iron Compounds

The following iron compounds were purchased from Sigma-Aldrich (schnelldorf, Germany): Iron(III) pyrophosphate (Product No. P6526), EDTA iron(III) sodium salt (Product No. 03650) and iron(II) fumarate (Product No. F5381). EDTA iron(III) sodium salt was suspended in ultrapure water to produce a stock solution [Fe] = 5 mM. Iron(III) pyrophosphate and iron(II) fumarate were dissolved in HCl (0.05 M). In Hutu-80 cells, the addition of FeEDTA stock solution to the medium caused a slight drop in pH from 7.25 to 7.15, a drop for ferrous fumarate from 7.25 to 6.75, and a drop for ferric pyrophosphate from 7.25 to 6.9 (measured immediately after iron addition).

### 2.2. Solubility Characterization of Iron Compounds

Iron in each iron-supplemented medium was fractionated into microparticulate, nanoparticulate, and soluble forms using centrifugation and ultrafiltration [29]. Samples were first centrifuged at 10,000× *g* for 5 min, with the resulting pellet representing the microparticulate Fe fraction. The supernatant was then ultrafiltered using a 3000 Da molecular weight cut-off membrane (10,000× *g*, 10 min) to separate the nanoparticulate and the soluble Fe fractions. Iron concentrations in the total sample, the supernatant, and the ultrafiltrate were quantified using inductively coupled plasma optical emission spectrometry (ICP-OES; JY 2000, Horiba Jobin Yvon, Stanmore, UK).

### 2.3. Dynamic Light Scattering Analyses

Dynamic Light Scattering (DLS) was performed to measure particle size distribution using an Anton Paar Litesizer 500 (Anton Paar GmbH, Graz, Austria) equipped with a 658 nm laser. Samples of a control medium (MEM + FBS 5%), a medium containing ferrous fumarate (0.5 mM Fe), and ferric pyrophosphate (0.5 mM Fe), 48 h after mixing, were analyzed in disposable cuvettes at 37 °C. The instrument was operated in automatic mode, which adjusts the scattering angle, acquisition time, and number of runs to optimize the signal and data quality. Particle size distributions were reported as intensity-weighted values, which show the relative contribution of particles (Rel. Frequency %) at a given size to the total scattered light intensity. Since the intensity of scattering light increases with particle diameter, larger particles contribute significantly to the intensity-weighted distribution. The Z-average hydrodynamic diameter was analyzed for each sample as a measure of the overall mean particle size.

### 2.4. Microwave Digestion and Atomic Absorption Spectroscopy (AAS)

The iron content of the stock solutions was quantified using atomic absorption spectroscopy (AAS). Briefly, 1 mL of each stock solution was diluted in 7 mL of ultrapure water, 1.75 mL of concentrated nitric acid (trace metal grade; Fisher Scientific, Loughborough, UK), and 0.35 mL of concentrated hydrochloric acid (trace metal grade; Fisher Scientific, Loughborough, UK) in Teflon microwave digestion vessels. Microwave digestion was performed using an Ethos Plus microwave lab station (Milestone, Sorisole, Italy) at 180 °C for 20 min. Following digestion, iron concentrations were measured with an Agilent 240/280 Series atomic absorption spectrometer (Santa Clara, CA, USA).

### 2.5. Cell Culture

Caco-2 (ATCC^®^ HTB-37) and Hutu 80 (ATCC^®^ HTB-40) were purchased from ATCC (Manassas, VA, USA). All these cell lines originate from human adenocarcinomas (either duodenal (Hutu-80) or colorectal) and are commonly used in mechanistic or screening studies. All the cell lines were maintained in MEM (Gibco™) and were supplemented with FBS (10%) and antimicrobial reagent Normocin™ (100 μg/mL; Invivogen, Toulouse, France). The growth medium was changed every second day (except for weekends), and the cells were passaged at approximately 80% confluence (trypsin-EDTA 0.05%; Gibco, Waltham, MA, USA). The cell medium was changed 3 times per week, and the cells were passaged at approximately 80% confluence.

### 2.6. Cell Experiments

The cells were seeded in 12-well plates (Corning, San Francisco, CA, USA) at a density 200,000 cells/well. Iron solutions were prepared fresh on the day of the experiment. An appropriate medium with FBS (5%) was used for the experiments, supplemented with iron solutions, except for the controls, at [Fe] = 0.5 mM. The supplemented medium was aspirated after 48 h of incubation, and the cells were washed with PBS. A RIPA buffer (Sigma Aldrich, Schnelldorf, Germany) coupled with Pierce protease and phosphatase inhibitors (Thermoscientific, Rockford, IL, USA) was used for cell lysis. The total protein content was analyzed with BCA assay. All the samples were tested with a Ferritin kit (DRG Diagnostics GmbH, Marburg, Germany).

### 2.7. ELISA Measurements of COX-2

Cellular levels of COX-2 (#DYC4198-2, R&D Systems, Minnesota, MN, USA) were quantified using enzyme-linked immunosorbent assay (ELISA) kits, following the manufacturers’ protocols. The measured enzyme levels were normalized to the total cellular protein content from each of the wells that were estimated using the BCA assay (#23225, Pierce™ BCA Protein Assay Kits, ThermoFisher, Waltham, MA, USA).

### 2.8. SDS-PAGE and Western Blot

Cell lysates were prepared using a Laemmli sample buffer that was supplemented with β-mercaptoethanol and heated at 95–100 °C for 5 min. Equal amounts of protein (5 μg) were loaded onto TGX gels (Bio-Rad, Hercules, CA, USA) and separated by SDS-PAGE using a Tris/Glycine/SDS running buffer at 180 V. The proteins were then transferred onto PVDF membranes using the Trans-Blot Turbo Transfer System (Bio-Rad). Unspecific binding to the membranes was blocked for 5 min at room temperature by a blocking buffer (EveryBlot, Bio-Rad). The primary antibody against rabbit phospho-NF-κB (Human Phospho-RelA/NFkB p65 (S536) Antibody, #MAB72261, R&D Systems) was diluted in the blocking buffer (0.5 μg/mL). The secondary antibody, goat anti-rabbit IgG (#1706515, Bio-Rad), was used at a final concentration of 1 μg/mL. Detection was performed using a luminol and peroxide substrate (Bio-Rad), and signal visualization was carried out using the ChemiDoc™ XRS+ system (Bio-Rad). Band intensities were quantified using Image Lab™ software version 3.0.1 (Bio-Rad).

### 2.9. Statistics

Statistical comparisons between treatment groups were performed using an unpaired two-tailed, non-parametric Mann–Whitney U test in Microsoft Excel, with *p*-values determined using exact table values. *p*-values smaller than or equal to 0.05 (*p* < 0.05) were considered statistically significant. The results are presented as mean values ± standard error of the mean (SEM) or standard deviation (sdev) from a minimum of three biological replicates per condition (*n* = 3). Spearman’s rank correlation coefficient (ρ) was used to assess correlations between variables, as the sample size can be considered small (3 × triplicates). Correlation analyses were performed using Python (version 3.10.12) with the scipy.stats module (1.14.0). ρ = 0 suggested the absence of any monotonic association, meaning that changes in the rank of one variable were not systematically associated with changes in the rank of the other (no correlation). ρ > 0 indicated a positive correlation; both variables’ ranks rise together in a monotonic fashion. ρ < 0 indicated a negative monotonic relationship between two variables.

## 3. Results

### 3.1. Solubility of Iron Compounds in Cell Culture Medium, MEM-FBS (5%)

The solubility of iron in the stock solutions (Fe 5 mM) and in the media supplemented with FBS (5%) at Fe = 0.5 mM was assessed (Table 1). Ferric pyrophosphate was soluble to 62.5% of the stocks, while only to 13.8% of the cell media. Ferrous fumarate was soluble to 100% of the stocks, but insoluble to 99.7% of the cell media. In contrast, ferric EDTA was soluble to 97% of both stocks and cell media. We also noted the presence of larger particles in the cell culture media (at 48 h) in the wells with ferric pyrophosphate and ferrous fumarate but not in controls or Ferric EDTA-treated cells, as observed at 200 times magnification in the microscope.

### 3.2. Particle Diameter in Iron-Supplemented Cell Culture Medium, MEM-FBS (5%)

**Figure 1:**
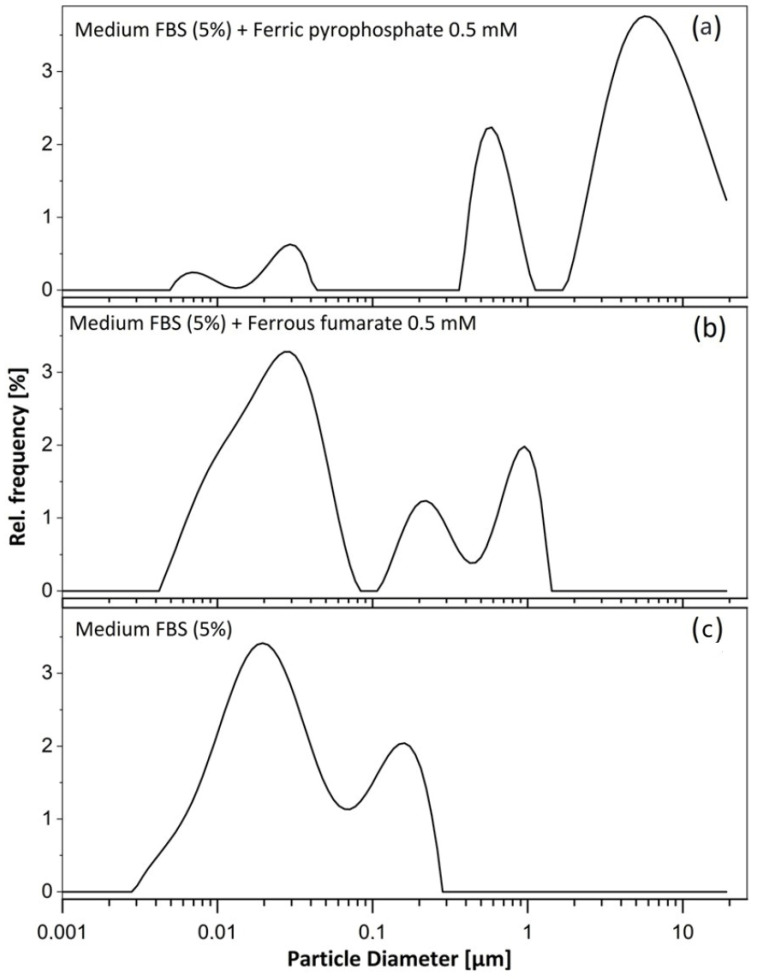
(**a**) MEM FBS, 5%, with ferric pyrophosphate (0.5 mM Fe; hydrodynamic diameter 2.129 µm); (**b**) MEM FBS, 5%, with ferrous fumarate (0.5 mM Fe; hydrodynamic diameter 0.036 µm); and (**c**) Intensity-weighted DLS size distributions of particulate matter in the control medium, MEM FBS, 5% (hydrodynamic diameter 0.03443 µm). The *y*-axis shows the relative frequency (%) of scattered light intensity for particles at a given hydrodynamic diameter (µm); The large micro-scale particles were only detected in sample (**a**). Before DLS analyses, the media samples were placed in an incubator at 37 °C for 48 h (allowing for air/CO_2_ mixing) to simulate cell culture conditions.

Dynamic light scattering (DLS) analysis confirmed the presence of larger particles in cell culture media containing ferric pyrophosphate (0.5 mM Fe, 48 h) and ferrous fumarate (0.5 mM Fe, 48 h) (Figure 1). In the control medium (MEM + 5% FBS; Figure 1), peaks were observed at 23 nm and 146 nm. In media supplemented with ferrous fumarate (Figure 1), a similar peak at ~25 nm was present, along with additional peaks at 237 nm and 866 nm. In media containing ferric pyrophosphate (Figure 1), peaks at 620 nm and 3.8 µm were detected, consistent with the formation of larger aggregates also observed by light microscopy. The Z-average hydrodynamic diameter was 2.129 µm for the ferric pyrophosphate sample, compared with a diameter of 0.036 µm for ferrous fumarate and a diameter of 0.034 µm for the control, indicating that there were micrometer-range particles in the medium containing ferric pyrophosphate and nanometer range particles in the ferrous fumarate and control media.

### 3.3. Ferric Pyrophosphate and Ferrous Fumarate Decreased Cell Survival

**Figure 2:**
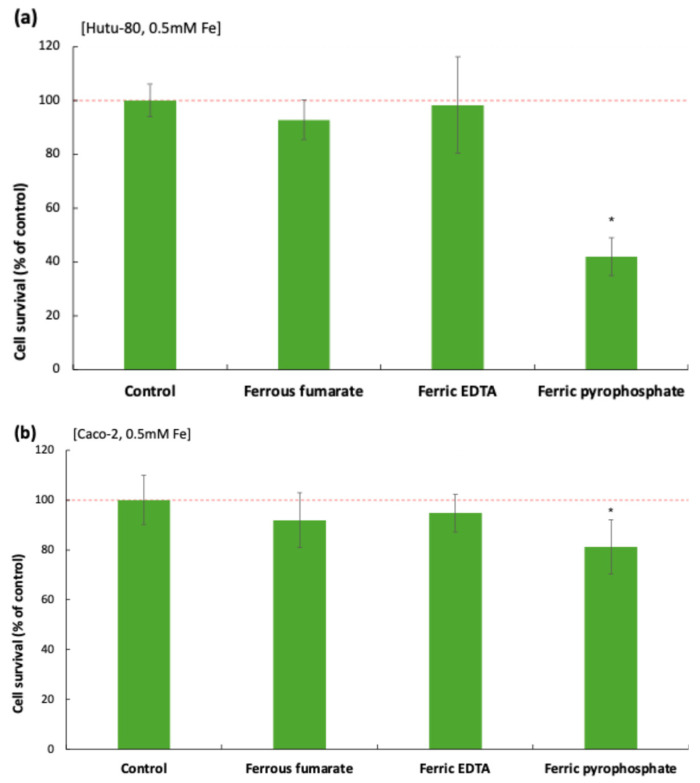
Cell survival (% of control) estimated from the change in total protein between iron-treated (0.5 mM, 48 h) cells and control cells: (**a**) Hutu-80 and (**b**) Caco-2. Data are means ± Sdev, *n* = 3 (nine replicates). A significant difference from control (*p* ≤ 0.05) is indicated with an asterisk (*). The red dashed line indicates the control level (100%).

Cell survival 48 h after exposure to iron treatments (0.5 mM Fe) was evaluated by measuring the total protein content per well, reflecting the density of remaining cells after washing (iron-treated vs. controls). Ferric pyrophosphate significantly reduced cell survival in both cell lines (Figure 2), and no significant changes were observed with ferrous fumarate or ferric EDTA in either cell line.

The inhibitory effect of pyrophosphate (0.5 mM, 48 h) was most pronounced in Hutu-80 cells (42% ± 7% remaining cells vs. control, *p* = 4.11 × 10^−5^) and smaller in Caco-2 cells (81% ± 11% remaining cells vs. control, *p* = 0.0019). There was a small effect (~7%) on cell survival in Hutu-80 cells treated with ferrous fumarate (93% ± 7% remaining cells vs. control, *p* = 0.04).

### 3.4. Induction of COX-2 and Intracellular Iron Load (Ferritin L)

**Figure 3:**
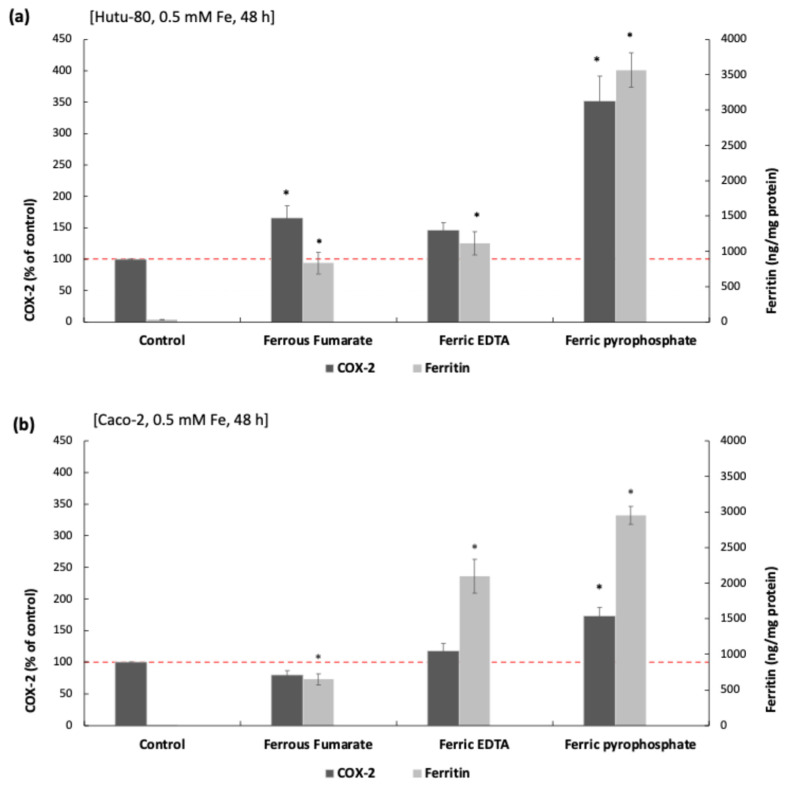
COX-2 (% of control, left-hand Y axes) and ferritin levels (ng/mg total protein, right-hand *Y*-axes) in (**a**) Hutu-80 and (**b**) Caco-2, measured with ELISA. Data are presented as means of nine cell replicates ± SEM, *n* = 3 separate occasions. Significant differences (*p* ≤ 0.05) in COX-2 levels from control cells, no added Fe, are indicated with asterisks (*). Baseline ferritin levels in untreated Caco-2 cells were 2 ± 1.3 ng/mg protein (mean ± Sdev) and were 34 ± 8 ng/mg protein (mean ± Sdev) in untreated Hutu-80. All iron treatments significantly increased ferritin levels from the control (*p* ≤ 0.05). The red dashed line indicates the level of COX-2 in controls (100%).

Ferric pyrophosphate treatment (0.5 mM Fe, 48 h) corresponding to an approximate oral iron dose of 170 mg in humans, given that a typical 60 mg elemental iron supplement can raise postprandial duodenal iron concentrations to about 200 µM after accounting for dilution in the stomach and duodenal lumen [29,30], resulted in a significant elevation of COX-2 protein levels in both Hutu-80 and Caco-2 cells (254% ± 37% increase vs. control, *p* = 4.11 × 10^−5^ and 78% ± 8%, *p* = 0.01, respectively; Figure 3a,b). Ferrous fumarate (0.5 mM Fe, 48 h) significantly increased COX-2 expression in Hutu-80 cells (62% ± 11%, *p* = 0.04). However, this effect was only accompanied by a small reduction (~7%) in cell survival, suggesting that the observed particle size (~237 nm) may be near the critical size threshold for inducing cytotoxicity.

To assess if the induction of COX-2 expression, following different iron compound exposure, was associated with intracellular iron accumulation, we analyzed the relationship between COX-2 protein and intracellular ferritin levels across the treatment groups.

Ferritin, an intracellular iron storage protein, serves as an indirect marker for cellular iron uptake [31,32], as its expression increases in response to elevated intracellular iron levels.

There was no significant correlation (Spearman’s rank correlation coefficient (ρ)) between ferritin levels and COX-2 in Hutu-80 cells. However, in Caco-2 cells, an inverse correlation between ferritin and COX-2 was observed after ferric pyrophosphate (ρ = −0.72, *p* = 0.03) and ferrous fumarate treatment (ρ = −0.67, *p* = 0.049). The induction of COX-2 was significantly correlated with a reduction in cell survival for the ferric pyrophosphate treatment in Hutu-80 cells (ρ = −0.68, *p* = 0.04). There was a weak negative correlation for the smaller ferric pyrophosphate effect in Caco-2 cells; however, it was not significant (ρ = −0.08, *p* = 0.8).

## 4. Discussion

As introduced earlier, iron absorption in the intestine is traditionally considered to be mediated by soluble forms, which rely on a reduction and uptake of ionic iron species, primarily through the divalent metal transporter DMT1 at the plasma membrane. However, recent evidence indicates that this classical uptake route does not operate in isolation. Insoluble iron particles, previously regarded as non-absorbable, can enter cells via endocytic mechanisms [1,9,10]. The co-utilization of these pathways for soluble and insoluble iron may create a synergistic effect, increasing overall iron availability. Indeed, insoluble iron imported through endocytosis has been associated with high bioavailability as observed in both human and murine studies, for example, evidence from a controlled human trial [33] and a preclinical murine model [34], respectively, shows that two insoluble iron compounds, Iron Hydroxide Adipate Tartrate (IHAT™) and Sucrosomial^®^ iron (destabilized ferric pyrophosphate, approximately 200 nm particle size) raise serum hemoglobin and ferritin concentrations to levels comparable with ferrous sulfate supplementation, thus contributing just as effectively to systemic iron pools.

The present study adds another layer of complexity to the issue of iron insolubility with the discovery that ferric pyrophosphate formed microscale particles in the cell culture medium, accompanied by cell death and elevated COX-2 expression. In addition, one of our preferred compounds, ferrous fumarate, induced COX-2 expression in Hutu-80 cells. This was unexpected because these two compounds differ markedly in their chemical profiles: ferrous fumarate is a salt with relatively high iron-ligand dissociation, whereas ferric pyrophosphate is a chelate with stronger ligand binding. However, a common denominator is that both are poorly soluble at duodenal pH, which we confirmed in this study.

The observation that microscale particles consistently caused an effect, and that larger nanoscale particles (~237 nm) may also have an impact, suggests proximity to a critical size threshold. This prompted us to include a smaller nanoscale iron compound, developed for iron fortification and supplementation, for comparison. We therefore conducted a pilot experiment with iron hydroxide adipate tartrate (IHAT™), a nanoparticulate iron compound (~2–5 nm), which is considerably smaller than the particles detected in MEM-FBS (5%). Under the same experimental conditions, IHAT™ did not induce COX-2 expression when compared with the control in Hutu-80 cells (Supplementary Figure S1), supporting the hypothesis that particle size plays a key role.

On another note, the higher COX-2 expression in response to ferric pyrophosphate observed in Hutu-80 cells compared with Caco-2 cells was accompanied by a more pronounced reduction in cell survival. Several factors may contribute to the lower resilience of Hutu-80 cells. Hutu-80 are of duodenal origin, whereas Caco-2 originate from the colon. Caco-2 cells are generally considered more resilient, as they more readily form tight junctions and polarize in comparison to Hutu-80. Although, in the present study, the cells were cultured for only 48 h (seeding density: 200,000 cells/3.5 cm^2^) and were just about confluent, as these features were not fully developed.

From a mechanistic perspective, the association between larger particles and COX-2 induction may involve reduced oxygen diffusion at the cell surface, favoring stabilization of hypoxia-inducible factors such as HIFs and HIF-2α. HIF-2α is a recognized transcriptional activator of COX-2 in intestinal and tumor tissues [35] and is also linked to increased iron uptake, for example, during hypoxia (e.g., [11]). This may explain the higher ratio of intracellular ferritin to COX-2 observed in cells with the strongest COX-2 response, particularly those treated with ferric pyrophosphate.

## 5. Conclusions

In conclusion, particle size appears to be a determinant for cell survival and iron-induced COX-2 expression during the iron supplementation of intestinal cells. While the simultaneous uptake of soluble and insoluble iron may optimize bioavailability, controlling particle size could reduce cell death and the induction of COX-2, as well as potentially other pro-inflammatory mediators. Moreover, the relationship between the iron dose and particle formation warrants consideration. In addition, it should be investigated whether COX-2 induction is limited to iron-containing particles and not triggered by any micro-sized particles.

These findings may have direct implications for human cell culture systems, both in mechanistic studies and in ex vivo applications for cell therapies. In a broader perspective, the findings should also be validated in vivo during dietary iron supplementation studies to evaluate if particle size can be associated with or influence intestinal inflammation.

## Figures and Tables

**Table 1:** Solubility data of iron compounds in the stock solutions and in the cell culture medium, MEM-FBS 5%. The data were collected by means of fractionation using centrifugation and ultrafiltration (<3 kD).

Iron Compound	Microparticulate Fe in Stock (%)	Nanoparticulate Fe in Stock (%)	Soluble Fe In Stock (%)	Microparticulate Fe in MEM-FBS (%)	Nanoparticulate Fe in MEM-FBS (%)	Soluble Fe in MEM-FBS (%)
Ferric pyrophosphate (HCl 50 mM)	9.5	28	62.5	1.9	84.3	13.8
Ferric EDTA	0	3.3	96.7	0.9	1.9	97.2
Ferrous fumarate (HCl 50 mM)	0	0	100	0.1	99.7	0.2

## Data Availability

The original contributions presented in this study are included in the article/Supplementary Material. Further inquiries can be directed to the corresponding author.

## Supplementary Materials

The following supporting information can be downloaded at https://www.mdpi.com/article/10.3390/biom16030388/s1. Figure S1: COX-2 levels in non-confluent Hutu-80 cells treated with the nanoparticulate iron compound iron hydroxide adipate tartrate (IHAT™).
