# Supplementary material for: Beyond Iron Solubility: Particle Size as a Determinant of Cell Survival and Iron-Induced COX-2 Expression in Human Intestinal Cells
Source: Biomolecules. 2026 Mar 5;16(3):388. doi: 10.3390/biom16030388 (PMC13024039; doi:10.3390/biom16030388)
Supplement: Supplementary file 1 [file biomolecules-16-00388-s001.zip › biomolecules-4090002-supplementary.pdf]

Supplementary figure S1.

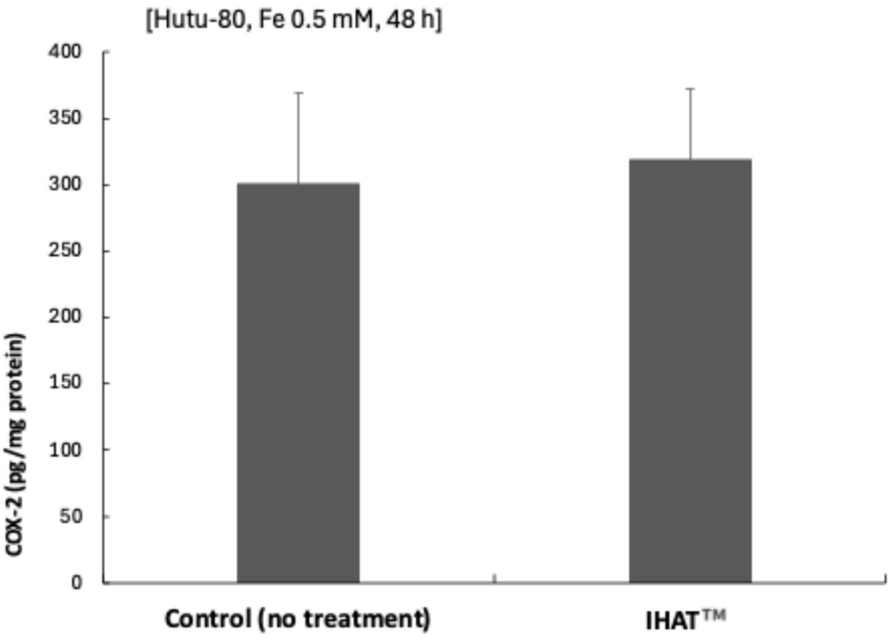

Supplemental figure S1. COX-2 levels normalized to total cell protein in non-confluent Hutu-80 cells treated with the nanoparticulate iron compound iron hydroxide adipate tartrate (IHAT™). Data are means +/- Sdev, n=2 in triplicates. The difference between treatment and control was not significant (p=0.2).
